# The development of novel ionic liquid-based solid catalysts and the conversion of 5-hydroxymethylfurfural from lignocellulosic biomass

**DOI:** 10.3389/fchem.2022.1084089

**Published:** 2022-12-02

**Authors:** Xiaofang Liu, Hangyu Luo, Dayong Yu, Zhengfei Pei, Zhuangzhuang Zhang, Can Li

**Affiliations:** Guizhou Provincial Key Laboratory for Rare Animal and Economic Insects of the Mountainous Region, College of Biology and Environmental Engineering, Guiyang University, Guiyang, China

**Keywords:** 5-hydroxymethylfurfural, ionic liquids, lignocellulosic biomass, cellulose, hemicellulose

## Abstract

Ionic liquids have attracted attention due to their excellent properties and potential for use as co-solvents, solvents, co-catalysts, catalysts, and as other chemical reagents. This mini-review focuses on the properties and structures of ionic liquids, the pretreatment of lignocellulosic biomass, and the development of novel ionic liquid-based solid catalysts for cellulose and hemicellulose derived HMF production.

## Introduction

As reported, lignocellulosic biomass is abundant (Zabed et al., 2017), has carbon–neutral properties, and is a sustainable and non-edible green feedstock (Hoang et al., 2021) that is a potential source material for the production of valuable biofuels and chemicals. The percentage of each constituent is determined by the wood/plant species, but, in general, is composed of cellulose (40%–50%), hemicellulose (25%–30%), and lignin (15%–20%), as well as small amounts of pectin, nitrogen compounds, and inorganic compounds (Kumar A. A. et al., 2020). The compound 5-hydroxymethylfurfural (HMF) is known as the “sleeping giant” of renewable intermediate chemicals, with derivatives that can be used in applications such as pesticides, medicines, and biofuel chemistry (Osatiashtiani et al., 2015; Le et al., 2022; Nasrollahzadeh et al., 2022). With current energy shortages and environmental pollution, it is critical that we seek green, sustainable, and alternative solutions.

## Properties and structures of ionic liquids

Ethyl ammonium nitrate, which is a liquid at ambient temperature and pressure, was the first described ionic liquid (IL) in 1914 (Walden, 1914; Angell et al., 2012). ILs have been widely identified as green substitutes for organic solvents based on their near-zero vapor pressures, high thermal stability and devisable polarity, hydrophobicity, and excellent capacity as solvents through modification of cations and anions (Zhang et al., 2014; Amarasekara, 2016). To the best of our knowledge, whether ILs can be recognized as green depends on approaches to synthesis and internal physico-chemical properties.

An IL can also be regarded as a salt, depending on whether its melting point is below 100°C, as salts consist of large and asymmetrical ions that typically have lower melting points (Berthod et al., 2017). Generally, the melting temperature, Tm, decreases with increased size, anisotropy, and internal flexibility of the ions; however, Tm increases with enhanced alkyl chain interaction (Weingärtner, 2008). Viscosity is one of the most significant material properties of ILs. High values for viscosity limit applications of ILs in various areas, since it reduces rate of reaction and molecule diffusion by forming a circulation barrier (Weingärtner, 2008). Tests of the thermal stability of ILs have shown that decomposition happens slowly at nearly 200°C (Berthod et al., 2017) in a process that hinges on the unique cation and corresponding anion association. The thermal stability of the imidazolium salts, along with the growth of the number of alkyl substitutions, has been demonstrated (Ngo et al., 2000). ILs involving linear side chains are more thermally stable than comparable branched monocationic ones (Xue et al., 2016). Halide anions can also decrease thermal stability to a certain extent. Generally, cations account for viscosity, melting point, and electrochemical stability, whereas anions are responsible for hydrogen bonding and miscibility with other solvents or water (Puripat et al., 2016).

**FIGURE 1:**
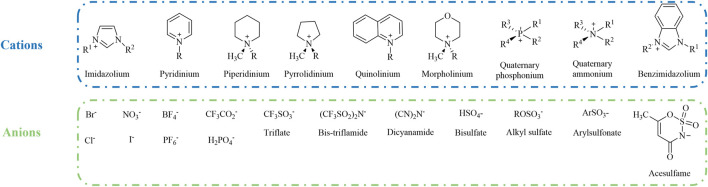
Commonly used cations and anions for ionic liquid combination.

The first-generation ILs defined dialkylimidazolium and alkylpyridinium as cations and metal halide (FeCl_4_^−^ and Al_2_Cl_7_^−^) as anions sensitive to water and air. Cations of quaternary ammonium and phosphonium containing dialkylimidazolium, alkylpyridinium, ammonium, and phosphonium, along with the classic anions tetrafluoroborate (BF_4_^−^) and hexafluorophosphate (PF_6_^−^) made up the second generation of ILs, which are not sensitive to either water or air. Unfortunately, slow hydrolysis of these anions with increased temperature leads to the production of hazardous and ecotoxic hydrogen fluoride (HF). From a green, sustainability perspective, second-generation ILs also show poor biodegradability, and are neither cost-effective nor green (Deetlefs and Seddon, 2010). Third-generation ILs are biodegradable cations and anions, and natural compounds containing choline, amino acids, or carbohydrates have been developed for IL production (Egorova et al., 2017). Widespread commercial application of ILs in various fields has been studied on account of these attributes (Figure 1).

Because of the above-mentioned characteristics, ILs have been identified as excellent solvents and catalysts for the synthesis of HMF from biomass and associated derivatives (Jiang et al., 2016; Li et al., 2018). Among IL cations, the mono-imidazole type has exhibited excellent performance. To facilitate the interaction between ILs and biomass or carbohydrates, di-/tri-cationic ILs with higher density and more hydrogen bonding were developed (Marullo et al., 2019; Rathod et al., 2019; Prasad et al., 2021).

## Catalytic transformation of lignocellulosic biomass in IL-based catalysts

**FIGURE 2:**
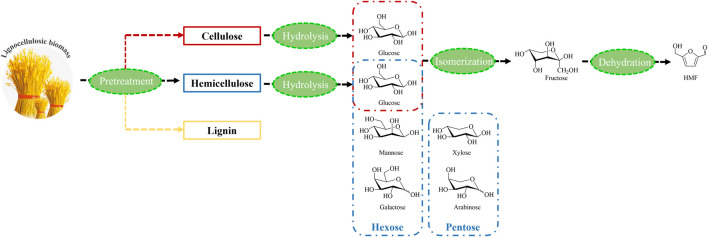
Different components of lignocellulosic biomass and the degradations.

Lignocellulosic biomass pretreatment is a significant process in the production of biofuels and value-added chemicals, the degradation of which is hindered by chemical properties, chemical structure, and microscopic complexity. Therefore, new approaches and reaction parameters are determined by attributes such as the degree of crystallization and polymerization of cellulose and the percentage of hemicellulose and lignin (Mood et al., 2013; Abraham et al., 2020) (Figure 2). Various ILs have been investigated to determine their effectiveness in lignocellulose dissolution and degradation to main compounds (Bian et al., 2014), although without the desired target HMF yield due to the multiple steps necessary to isolate HMF from raw materials.

Kahani et al. (2017) synthesized N-allyl-N-methylmorpholinium acetate ([AMMorph][Ac]) and successfully introduced it for the pretreatment of rice straw. Compared to the most efficient solvent imidazolium liquids, the morpholinium liquids are less toxic and less expensive, while providing glucose yields of 98.4 ± 1.3% using mixtures of [AMMorph][Ac]-DMSO (70:30, v/v) and N-methylmorpholine-N-oxide (NMMO), [Bmim][Ac], and NaOH at 120°C. Furthermore, using DMSO as a co-solvent could minimize IL usage and enhance pretreatment efficiency by reducing viscosity (Kahani et al., 2017).

Use of a novel integrated –SO_3_H functionalized IL catalyst [IL-SO_3_H][Cl] and nickel sulfate (NiSO_4_.6H_2_O) co-catalyst resulted in a maximum glucose conversion of 99.92%, with 21.80% HMF yield when incubated at 175°C for 1.5 h in the aqueous phase (Kumar K. et al., 2020). According to the pseudo-first-order kinetic equations, the activation energy (*E*_*a*_) and pre-exponential factor (*A*) was confirmed to be 47.45 kJ/mol and 7.9 × 10^3^ min^−1^, respectively, for conversion of glucose into HMF. The research demonstrated an effective synergistic effect of the IL catalyst and Lewis acidic co-catalyst in clean synthesis of HMF from waste biomass derived glucose, providing a promising pathway for the preparation of vital platform chemicals and renewable fuels.

## Conversion of cellulose with IL-based catalysts

The synthesis of HMF derived from cellulose involves several steps: depolymerization, hydrolysis, isomerization and, ultimately, dehydration (Tyufekchiev et al., 2018; Zhang et al., 2018a; Zhang et al., 2018b; Naz et al., 2021). For different processes, catalyst properties vary depending on the reactions.

Excessive Brønsted acid or Lewis acid can accelerate the side reactions that produce by-products. Therefore, the “tailor-made” development of satisfactory catalyst processing with the appropriate ratio of Lewis acid to Brønsted acid is crucial for increasing HMF yield. In addition, allylimidazole-type ILs have strong advantages in cellulose dissolution (Liu et al., 2012).

At the beginning of the instantiation phase, metallic ILs (i.e., Cr([PSMIM]HSO_4_)_3_ and CuCr([PSMIM]SO_4_)_5_) were designed and applied to the cellulose-HMF system (Zhou et al., 2013). Cr([PSMIM]HSO_4_)_3_ demonstrated higher catalytic performance in the production of HMF, with 53% yield ascribed to the bifunctionality and higher Brønsted acidity at 120°C.

Liu et al. (2022) prepared a series of reactions with different proportions of Brønsted acid and Lewis acid ILs for the degradation of cellulose to produce HMF. Among these, [(HSO_3_-P)_2_im]Cl⋅ZnCl_2_ exhibited excellent catalytic performance, with an HMF yield of 65.66% at 140°C for 3 h. This study facilitated directional optimization of the catalyst. The quantum chemical calculation method for molecular design was used to predict the catalytic effect (different ratios of Brønsted acid to Lewis acid) and investigate the catalytic mechanism. Therefore, the solvation model density (SMD) model was proposed in combination with Frontier orbital theory. In addition, cellulose degradation experiments were performed to verify the simulation results and inform discussion of the catalytic mechanism (Liu et al., 2022).

## Conversion of hemicellulose by IL-based catalysts

As the second most predominant component of lignocellulosic biomass, hemicellulose is composed of pentoses like xylose, arabinose, and hexose, including glucose, mannose, galactose, and the amorphous polymer xylan (Ruiz et al., 2013). There are few developments in the production of HMF derived from mannose and galactose. Researchers demonstrated that mannose is predominantly isomerized to fructose, which can be efficiently converted into HMF, while galactose primarily isomerizes to tagatose, which is the C-4 epimer of fructose and weaker than fructose in yielding HMF (van Putten et al., 2013).

When lignocellulosic biomass was employed as raw material, cellulose with higher HMF yield than hemicellulose was preferentially chosen for the synthesis of HMF, as the research was aimed at improving the conversion efficiency of cellulose (Menegazzo et al., 2018). Considering the complex components involved, the conversion conditions for HMF are difficult to control.

## Conclusion and outlook

HMF yield close to 100% will eventually be achieved by adjusting the approach to isolation of the compound from feedstock and optimizing IL-based catalyst reaction conditions. This cost-effective, green, sustainable catalyst system, which inhibits by-products, is easily functionalized, and has no adverse environmental impact, will lead to significant advances in future industrial-scale HMF production. Density functional theory (DFT) and molecular dynamics should be applied in biomass conversion to aid in the development of reliable reaction pathways.
